# Metagenomic insights into antibiotic resistance genes and virulence factors in sediments of river Yamuna

**DOI:** 10.3389/fmicb.2026.1938385

**Published:** 2026-08-28

**Authors:** Ajaya Kumar Rout, Partha Sarathi Tripathy, Sujata Dey, Neelesh Kumar, Ganesh Kumar, Pranaya Kumar Parida, Ashutosh Singh, Budheswar Dehury, Pramod Kumar Pandey, Bijay Kumar Behera

**Affiliations:** 1 College of Fisheries (Datia), Rani Lakshmi Bai Central Agricultural University, Jhansi, India; 2 ICAR-Central Inland Fisheries Research Institute, Barrackpore, India; 3 College of Agriculture, Rani Lakshmi Bai Central Agricultural University, Jhansi, India; 4 Department of Bioinformatics, Manipal School of Life Sciences, Manipal Academy of Higher Education, Manipal, India; 5 National Fisheries Development Board, Department of Fisheries, Government of India, Hyderabad, India

**Keywords:** AMR, metagenomics, microbial diversity, river Yamuna, sediment, virulence factors

## Abstract

Riverine sediments serve as critical reservoirs of microbial diversity and functional genes, reflecting both natural ecological processes and anthropogenic impacts. In the present study, we employed a shotgun metagenomic approach to investigate microbial community composition, antimicrobial resistance (AMR) genes, and virulence factors in sediments collected from three environmentally distinct locations of the Yamuna River near Agra, India, representing BSA, TGY, and YEA. The sediment DNA was subjected to high-throughput Illumina sequencing, followed by quality control, assembly, and open reading frame prediction. Taxonomic classification and diversity analyses were performed using MEGAN6 and R-based statistical tools, while AMR genes were identified from predicted metagenomic proteins using the Resistance Gene Identifier (RGI) against the CARD database, with high-confidence perfect and strict hits retained; ARGs were interpreted independently of species-level host assignment. Virulence factors were assessed through presence–absence profiling of functionally relevant gene categories. The results revealed pronounced spatial heterogeneity in microbial communities, with increasing taxonomic diversity, functional complexity, and evenness from BSA to TGY and YEA. TGY and YEA composite samples showed greater observed representation of high-confidence AMR gene predictions spanning multiple drug classes and resistance mechanisms, alongside a diverse repertoire of virulence-associated genes linked to motility, adhesion, and secretion systems. In contrast, the BSA site harbored a comparatively simpler resistome and virulome. Overall, this study highlights Yamuna River sediments as important reservoirs of resistance and virulence determinants and underscores the need for long-term genomic surveillance to inform risk assessment, pollution control, and sustainable river management strategies.

## 1 Introduction

Metagenomics represents a transformative approach in microbiology, enabling direct analysis of genetic material from environmental samples to comprehensively characterize uncultured microbial communities (Behera et al., 2021; Rout et al., 2025). Unlike traditional culture-dependent methods, which capture < 1% of microbial diversity due to the uncultivability of most environmental microbes, metagenomics provides a culture-independent framework for elucidating taxonomic composition, functional potential, and ecological dynamics across diverse habitats, including soils, aquatic systems and host-associated microbiomes (Nam et al., 2023; Sodhi et al., 2021). This methodology has revolutionized multiple fields such as ecology, human health, biotechnology, and environmental microbiology by facilitating high-resolution profiling of community structure, antibiotic resistance genes (ARGs), potential plastic, xenobiotic biodegradation pathways, novel enzyme discovery, and microbial responses to anthropogenic stressors (Das et al., 2020; Rout et al., 2024a; Samson et al., 2019). Metagenomic and bioinformatic analyses have significantly advanced the identification of beneficial microbiome, bacteriophages, and bioremediating bacteria from sediments of the rivers Ganga and Yamuna with potential applications (Behera et al., 2020a,b, 2021). Furthermore, multiple studies employing metagenomic approaches have characterized microbial community composition and elucidated functional potential in various river sediment ecosystems, providing critical insights into their ecological roles and contaminant degradation capabilities (Abia et al., 2018; Reddy and Dubey, 2019). Riverine microbial communities in South Asian rivers are highly dynamic and respond to interacting hydrological, physicochemical, and anthropogenic controls. In the Ganges–Yamuna river system, variations in water flow, seasonal conditions, physicochemical characteristics, and contaminant inputs can influence microbial diversity, community structure, and functional activity. Microbial assemblages may therefore provide useful indicators of environmental disturbance and anthropogenic contamination, while also contributing to nutrient cycling, organic matter transformation, and ecosystem functioning. Recent synthesis of the Ganges and Yamuna microbiomes has emphasized the importance of integrating hydrological and ecological processes when interpreting spatial patterns in riverine microbial communities (Chaturvedi et al., 2024).

Microbes, including bacteria, fungi, viruses, and other microscopic organisms, play a crucial role in the functioning and balancing of ecosystems. Sediments near riverbanks provide a distinct habitat for microbial communities that contribute significantly to the functioning of river ecosystems. These microbial assemblages perform various metabolic functions that are crucial for biogeochemical and nutrient cycling, biophysical processes, and energy flow within river ecosystems (Zhang et al., 2016). The microbial load in river sediments, along with their responses to environmental cues and anthropogenic disturbances, can serve as indicators of the health and quality of river ecosystems. Anthropogenic activities, such as industrial discharge, sewage pollution, agricultural runoff, and improper waste management, can introduce pathogens and contaminants into river ecosystems (Rajeev et al., 2021; Rout et al., 2022).

The river Yamuna originates from the Yamunotri Glacier in the lower Himalayas and flows through several states, including Uttarakhand, Himachal Pradesh, Haryana, Delhi, and Uttar Pradesh, before joining the Ganga River at Allahabad (Prayagraj). The Yamuna River holds great mythological and historical importance in India. Despite being the longest tributary of the Ganga, the Yamuna is severely polluted, significantly degrading its water quality (Mittal et al., 2019). The river Yamuna has faced severe issues, including gradual flow restriction and higher levels of pollution, especially as a result of the discharge of city sewage and industrial effluent from New Delhi, Agra and its neighboring areas (Raghav et al., 2024; Sodhi et al., 2021). The high concentrations of organic substances and antimicrobial agents, along with the low flow rate, acted as a natural bioreactor for the development of bacteria resistant to antibiotics (Rout et al., 2024b). The Central Pollution Control Board (CPCB) of India has identified 18 major drains and their impact on the water quality of the river Yamuna. Untreated discharge from urban runoff, including fecal, hospital, domestic, and industrial waste, is a major contributor to pollution of the Yamuna River. These sources introduce a range of pollutants into the river, including organic matter, toxic chemicals, and heavy metals. Low levels of dissolved oxygen (DO) and very high levels of biochemical oxygen demand (BOD) are indicators of the deteriorating water quality of river Yamuna (Mittal et al., 2019). The densely populated city of Delhi, located on the banks of the Yamuna River, relies heavily on it as a vital water source for its various needs. The Yamuna River, especially the stretch that passes through Delhi, receives substantial amounts of domestic and industrial sewage from numerous industries along its banks (Parida et al., 2022).

The occurrence of antibiotic resistance genes (ARGs) in metagenomic data from Yamuna River sediments is critical for assessing potential risks to ecosystem integrity and to the large human populations that depend on this river for domestic, recreational, and cultural purposes (Das et al., 2020). A detailed understanding of the prevalence, diversity, and abundance of VFs provides key insights into the pathogenic potential of sediment-associated microbial communities and their implications for public health (Rout et al., 2024b). These determinants include genes involved in toxin production, adhesion, invasion, immune evasion, and other pathogenicity-related mechanisms, and their presence suggests that sediment-dwelling microbes may exhibit harmful traits or cause disease when exposed through appropriate routes. Similarly, profiling the distribution, diversity, and molecular mechanisms of ARGs in sediment microbiomes yields valuable information on environmental resistome reservoirs and the potential for horizontal gene transfer to clinically relevant bacteria. Identifying ARGs and VFs in its sediments is essential for quantitative health risk assessment and for designing evidence-based surveillance and intervention strategies. Moreover, the enrichment of ARGs and VFs can alter microbial community structure and function, disrupt species interactions, and perturb key biogeochemical processes, thereby influencing the ecological balance and resilience of the riverine ecosystem (Rout et al., 2023).

In this study, we employed shotgun metagenomics which reveals microbial community dynamics and functional adaptations in Yamuna River sediments across pollution gradients at Agra, India. Diverse anthropogenic pollutants drive selection for genetic mechanisms enabling organic matter degradation, heavy metal resistance, and xenobiotic catabolism, with comprehensive profiling of antibiotic resistance genes (ARGs) and virulence factors across sites. Functional predictions identify metabolic capabilities of ARG-harboring taxa, providing critical data for developing targeted bioremediation strategies and evidence-based policies to restore aquatic biodiversity and sustain riverine ecosystem services.

## 2 Materials and methods

### 2.1 Sample collection

Sediment and water samples were collected from three sites along the Yamuna River near Agra, Uttar Pradesh, India: Balkeshwar Shivpuri Agra (BSA; 27.221461°N, 78.03204°E), Taj Ganj Yamuna (TGY; 27.175609°N, 78.040207°E), and Yamuna Expressway Agra (YEA; 27.179351°N, 78.120992°E). Sampling occurred between 08:30 and 10:30 h in March 2021. At each site, composite sediment samples (∼500 g, derived from five subsamples spaced ∼250 m apart) and water samples (500 mL) were collected in sterile, autoclaved amber glass bottles and plastic bags, respectively. The five subsamples collected at each location were pooled to generate a single composite sediment sample per site. Thus, the five subsamples were not treated as independent biological replicates, and the final metagenomic dataset comprised one independent composite sediment sample from each of the three locations (*n* = 1 per site). Accordingly, downstream taxonomic, diversity, AMR, and virulence-factor comparisons among locations were interpreted as descriptive/exploratory comparisons rather than inferential statistical tests of site-level differences. Physicochemical parameters and heavy metals of water at different sampling sites are provided in (Supplementary Table 1). On-site pH (Hanna Instruments, USA) and temperature (MT-222 Digiflexi, Dr. Morepen, India) measurements were recorded. Samples were transported at 4°C and stored at -20°C prior to processing.

### 2.2 Genomic DNA isolation, library preparation and sequencing

Total genomic DNA was extracted from the sediment samples using the XpressDNA Soil Kit (MagGenome, USA) following the manufacturer’s instructions. DNA quality and integrity were assessed by electrophoresis on a 1% agarose gel, while DNA concentration and purity were evaluated using a NanoDrop spectrophotometer (Thermo Scientific, USA). DNA purity was assessed based on the A260/A280 ratio, with a target range of 1.8–2.0, and DNA concentration was recorded for each extract. DNA extracts yielding ≥ 1 μg total DNA and meeting the predefined purity and integrity criteria were considered suitable for library preparation. Extracted DNA was examined for the presence of high-molecular-weight genomic DNA and evidence of substantial degradation before proceeding to library preparation.

### 2.3 Sequence analysis and taxonomic classification

Raw reads were quality-filtered to remove adapter sequences, low-quality bases, and Homo sapiens contaminants using Bowtie2-based mapping. Taxonomic classification was performed using MEGAN6 with the lowest common ancestor (LCA) algorithm (Beier et al., 2017), against NCBI-nr database. The resulting taxonomic profiles were used to generate genus- and species-level abundance matrices for downstream community composition and diversity analyses.

### 2.4 Prediction of AMR genes

The prediction of AMR genes was performed using the Resistance Gene Identifier (RGI) tool (Kwatra, 2021) against the Comprehensive Antibiotic Resistance Database (CARD). Protein sequences predicted from the metagenomic assemblies of the three Yamuna River locations (BSA, TGY, and YEA) were analyzed using RGI under the specified default parameters. AMR determinants were identified based on sequence homology and curated resistance models within the CARD framework; therefore, species-level taxonomic assignment was not a prerequisite for ARG identification. RGI outputs were filtered to retain high-confidence perfect and strict matches. Identified AMR genes were categorized according to resistance mechanisms, antibiotic classes, and gene families. Because many metagenomic sequences could not be resolved to species level, ARG detection was interpreted independently of species-level taxonomic assignment, and specific host attribution of individual ARGs was not inferred unless supported by sufficient taxonomic resolution. For quantitative comparison of AMR genes among the three metagenomic samples, quality-filtered reads were mapped to the predicted AMR gene sequences identified by RGI. The number of reads assigned to each AMR gene was normalized for both gene length and sequencing depth and expressed as reads per kilobase per million mapped reads (RPKM). For each gene, RPKM was calculated as the number of mapped reads divided by gene length in kilobases and the total number of mapped reads in millions. Gene-level RPKM values were subsequently summarized by resistance mechanism and antibiotic class for comparative visualization among BSA, TGY, and YEA. The heatmaps representing the relative abundance and distribution of predicted AMR genes across BSA, TGY and YEA sampling locations were generated using the RGI visualization pipeline. The heatmap was constructed from normalized AMR gene counts and hierarchically clustered to reveal similarities and differences in AMR profiles across the three locations.

### 2.5 Quantification and normalization of ARG abundance

To distinguish ARG detection confidence from ARG abundance, quantitative abundance estimates were generated independently of RGI hit-confidence categories. Quality-filtered metagenomic reads were mapped against the AMR gene sequences identified from the corresponding assemblies. Gene-specific read counts were normalized for gene length and sequencing depth and reported as RPKM. For each ARG, RPKM was calculated as: RPKM = (number of reads mapped to the ARG × 10^9^)/(ARG length in bp × total mapped reads). The resulting quantitative abundance matrix was used to generate heatmaps and summarize ARG abundance by resistance mechanism and antibiotic class. Perfect and strict RGI hits were retained as annotation-confidence categories and were not interpreted as quantitative abundance measures.

### 2.6 Quantification of virulence-factor genes

Virulence-factor genes were quantified using the same read-mapping and normalization strategy applied to ARGs. Quality-filtered metagenomic reads were mapped to the nucleotide sequences of identified virulence-factor genes, and gene-specific read counts were normalized for gene length and sequencing depth. Quantitative abundance was expressed as RPKM, while presence–absence information was retained separately to describe gene detection. Thus, VF detection status and quantitative abundance were treated as distinct measures.

### 2.7 Statistical data analysis

Metagenomic data were analyzed to characterize microbial community composition, diversity, antimicrobial resistance (AMR), and virulence-associated gene profiles across the three sampling locations (BSA, TGY, and YEA). Following quality filtering and taxonomic classification using the MEGAN6 LCA algorithm against the NCBI-nr database, genus-level abundance matrices were generated for downstream diversity analyses. Alpha diversity was assessed using Observed species richness, Shannon diversity, and Simpson diversity indices using the vegan package in R. Beta diversity was evaluated using Bray-Curtis dissimilarity and visualized using principal coordinates analysis (PCoA). Taxonomic abundance profiles were visualized to compare the microbial community composition among the three sampled locations. AMR genes were identified using the Resistance Gene Identifier (RGI) against the Comprehensive Antibiotic Resistance Database (CARD), and their distribution was summarized according to resistance mechanisms and antibiotic classes. Virulence-associated genes were evaluated using presence–absence profiles across the sampling locations. Because only one composite sediment sample was available per location, these analyses were interpreted as descriptive and exploratory comparisons rather than inferential statistical tests of differences among locations.

## 3 Results

### 3.1 Sample information and descriptive sequence statistics

High-throughput metagenomic sequencing generated large, high-quality datasets at all three Yamuna River sampling locations, with notable variation in sequencing depth and assembly output among sites (Table 1). The BSA sample produced over 38.3 million reads, corresponding to approximately 5.75 Gb of sequence data, with more than 93% of reads exceeding Q20 quality scores. Assembly of these reads resulted in approximately 2.73 million contigs, the majority of which were longer than 150 bp. Gene prediction identified over 3.08 million open reading frames (ORFs), of which roughly 2.19 million exceed 150 bp. For TGY, sequencing yielded approximately 43.6 million reads totaling 6.54 Gb, with the highest proportion of high-quality reads (> 96% Q20) among the samples. Assembly produced more than 4.35 million contigs, predominantly above 150 bp. A total of 4.82 million ORFs were predicted from the assembly, of which approximately 3.38 million met or exceeded the 150 bp length threshold. The YEA dataset showed the greatest sequencing depth, comprising approximately 46.5 million reads and 6.98 Gb of sequence data, with > 95% of reads passing the Q20 quality filter. Assembly yielded nearly 5.0 million contigs, most of which were longer than 150 bp. Gene prediction yielded over 5.54 million ORFs, of which approximately 3.88 million were longer than 150 bp.

**TABLE 1:** Sequencing and assembly statistics conducted on three sediment samples collected from the river Yamuna at Agra in Uttar Pradesh.

Location	Sequencing depth (million reads)	Total bases (Gb)	High-quality reads (%)	Assembled contigs (total)	Contigs ≥ 150 bp	Contigs < 150 bp	Predicted ORFs (total)	ORFs ≥ 150 bp
BSA	38.34	5.75	93.50	2,731,848	2,681,310	50,538	3,083,326	2,186,940
TGY	43.59	6.54	96.50	4,357,687	4,275,139	82,548	4,821,175	3,380,076
YEA	46.54	6.98	95.50	4,960,271	4,874,276	85,995	5,543,201	3,883,324

### 3.2 Taxonomic abundance of microbial taxa

**FIGURE 1:**
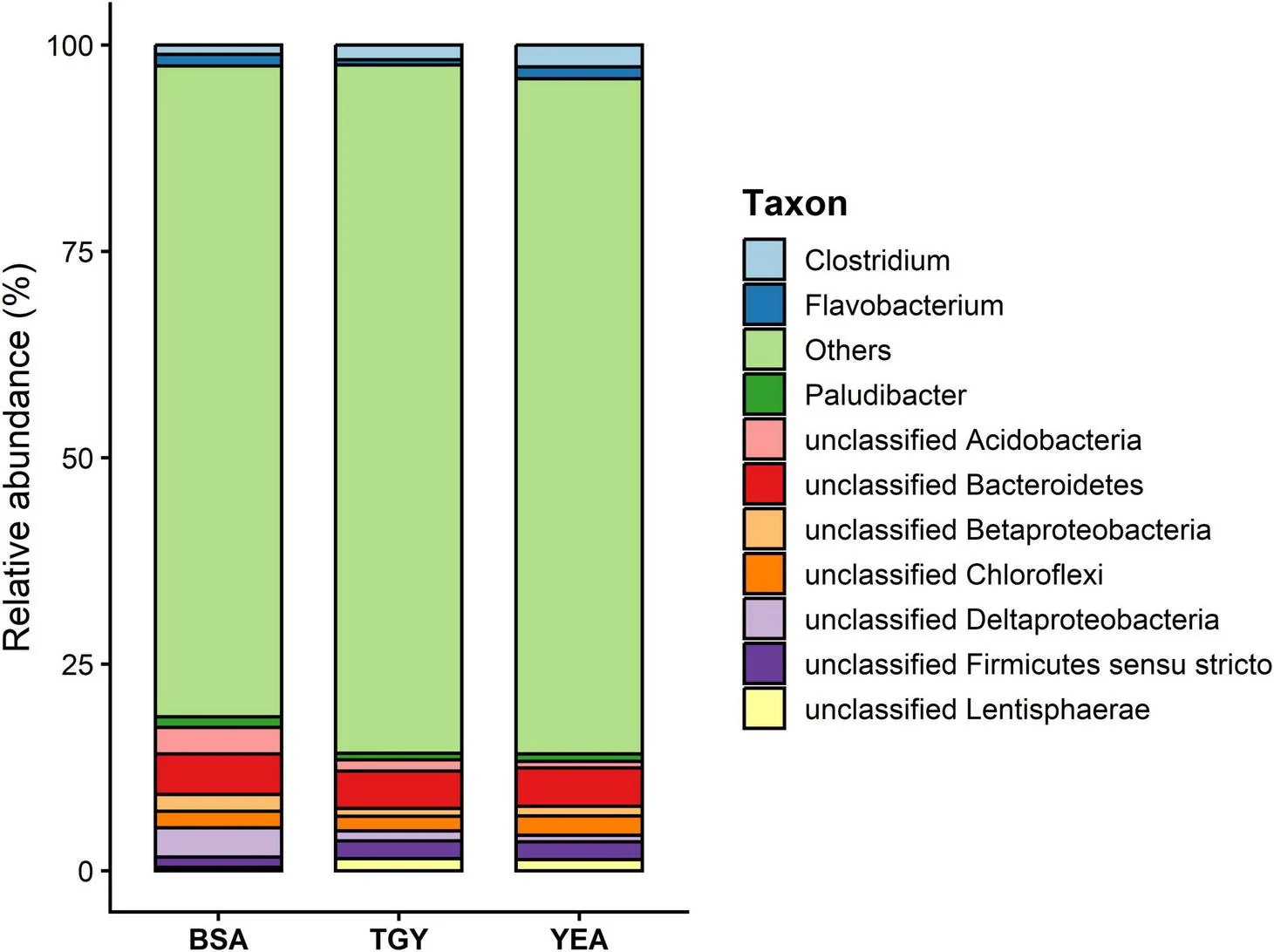
Genus-level taxonomic composition of microbial communities in Yamuna River sediments. Stacked bar plots show the relative abundance (%) of dominant and unclassified bacterial genera detected at the three sampling locations, i.e., BSA, TGY, and YEA. Taxa are grouped into major classified genera (*Clostridium*, *Flavobacterium*, *Paludibacter*) and unclassified bacterial groups, with low-abundance genera collectively represented as “Others.”

The taxonomic profiling of microbial communities at the genus level revealed distinct differences in relative abundance patterns among the three Yamuna River sampling locations (Figure 1).

At BSA, the microbial community showed a higher relative contribution of unclassified bacterial groups, particularly Bacteroidetes and Acidobacteria, compared to the other sites. Minor but consistent contributions from Deltaproteobacteria, Chloroflexi, and Firmicutes were also observed. Among the classified genera, Paludibacter was present at low abundance, while Flavobacterium and Clostridium contributed marginally to the overall community structure.

The TGY site exhibited a slightly altered taxonomic profile, with a relative reduction in Acidobacteria and an increase in Firmicutes and Lentisphaerae compared to BSA. The contribution of Bacteroidetes remained substantial, while Paludibacter was detected at low abundance. Flavobacterium and Clostridium were consistently present but contributed only a small fraction of the total community.

At YEA, the microbial community composition was broadly similar to that of TGY but showed subtle differences in relative abundances. Bacteroidetes and Betaproteobacteria constituted a noticeable fraction of the community, while Chloroflexi and Deltaproteobacteria were also present. The relative contributions of Clostridium and Flavobacterium remained low but consistent, and Paludibacter remained represented at low levels.

### 3.3 Diversity analysis

#### 3.3.1 Alpha diversity

**FIGURE 2:**
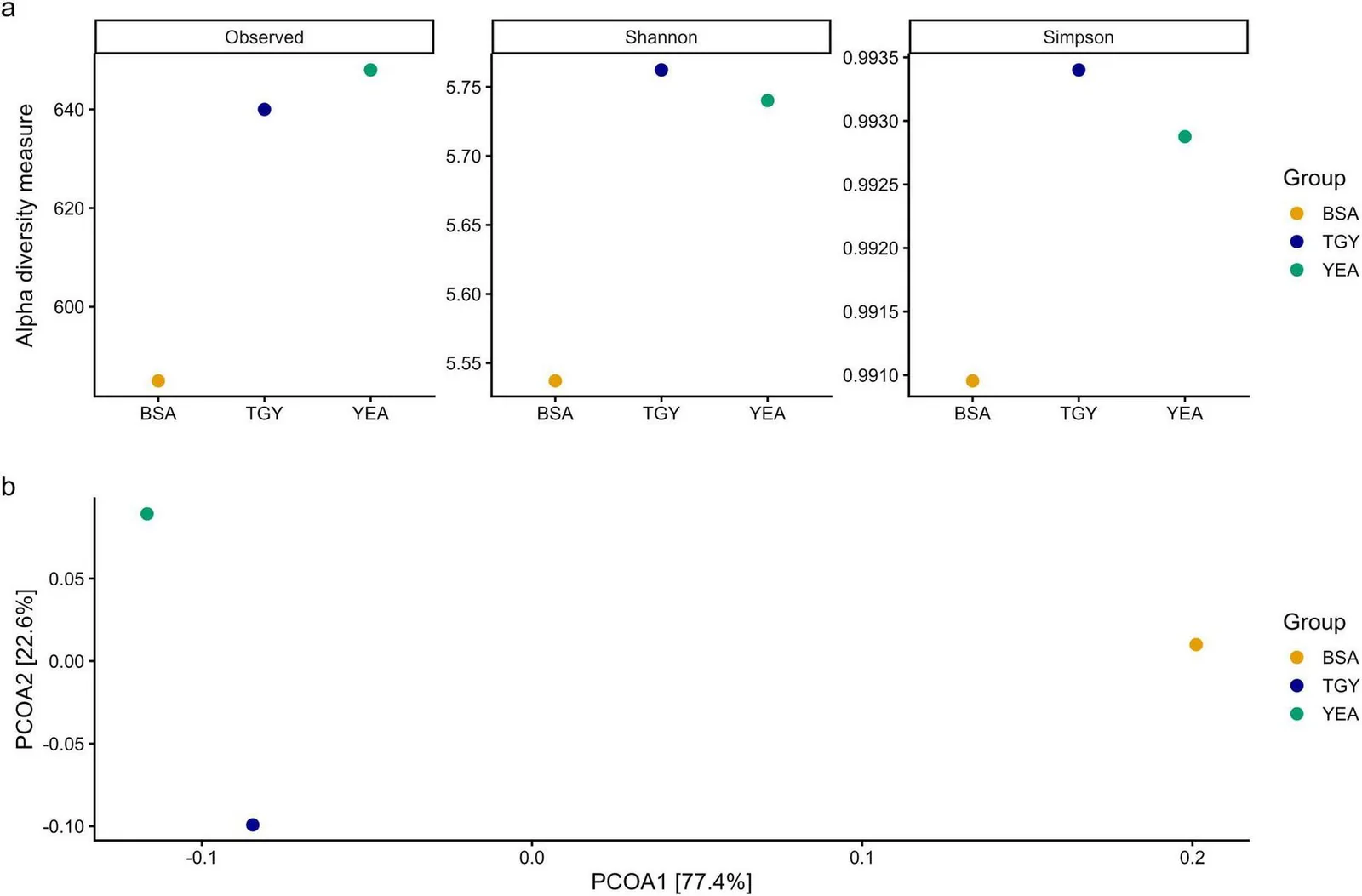
Exploratory alpha- and beta-diversity profiles of microbial communities across Yamuna River sampling locations. **(a)** Observed richness, Shannon diversity, and Simpson diversity for the single composite sediment sample from each location (BSA, TGY, and YEA). **(b)** Bray-Curtis dissimilarity visualized using principal coordinates analysis (PCoA). Each point represents one site-level composite sample; therefore, the figure illustrates descriptive differences among the three sampled locations rather than within-site variation or statistically tested site effects. The first principal coordinate (PCoA1) explains 77.4% of the total variation, while the second coordinate (PCoA2) explains 22.6%.

Descriptive alpha-diversity profiles showed differences in microbial richness and evenness among the three Yamuna River sampling locations (Figure 2a). The Observed species richness was lowest at BSA, higher at TGY, and highest at YEA, showing an apparent increase in the number of detected taxa from BSA to YEA. Consistent with the observed richness, the Shannon diversity index showed the lowest value at BSA, corresponding to the lowest observed community complexity among the three samples, while higher values were recorded at TGY and YEA. TGY exhibited the highest Shannon index, followed closely by YEA, indicating greater taxonomic diversity and a more even distribution of taxa at these two sites compared to BSA. The Simpson diversity index, which emphasizes dominance and evenness, also followed a similar trend. BSA displayed the lowest Simpson index, whereas TGY showed the highest value, with YEA slightly lower but still markedly higher than BSA. The high Simpson indices at TGY and YEA indicate highly even communities with limited dominance by a few taxa.

#### 3.3.2 Beta diversity

Exploratory beta-diversity analysis based on Bray–Curtis dissimilarity and principal coordinates analysis (PCoA) showed separation among the three site-level metagenomic profiles (Figure 2b). PCoA1 explained 77.4% of the variation, while PCoA2 accounted for 22.6%. The three composite sediment samples occupied distinct positions in the ordination space, indicating differences in their overall microbial community composition. BSA was positioned separately along PCoA1, whereas TGY and YEA were separated primarily along PCoA2. Because only one composite sample was available per location, these patterns should be regarded as exploratory site-level differences rather than evidence of statistically supported separation among populations or sampling locations.

### 3.4 AMR genes across the three locations

#### 3.4.1 AMR genes based on drug classification

**FIGURE 3:**
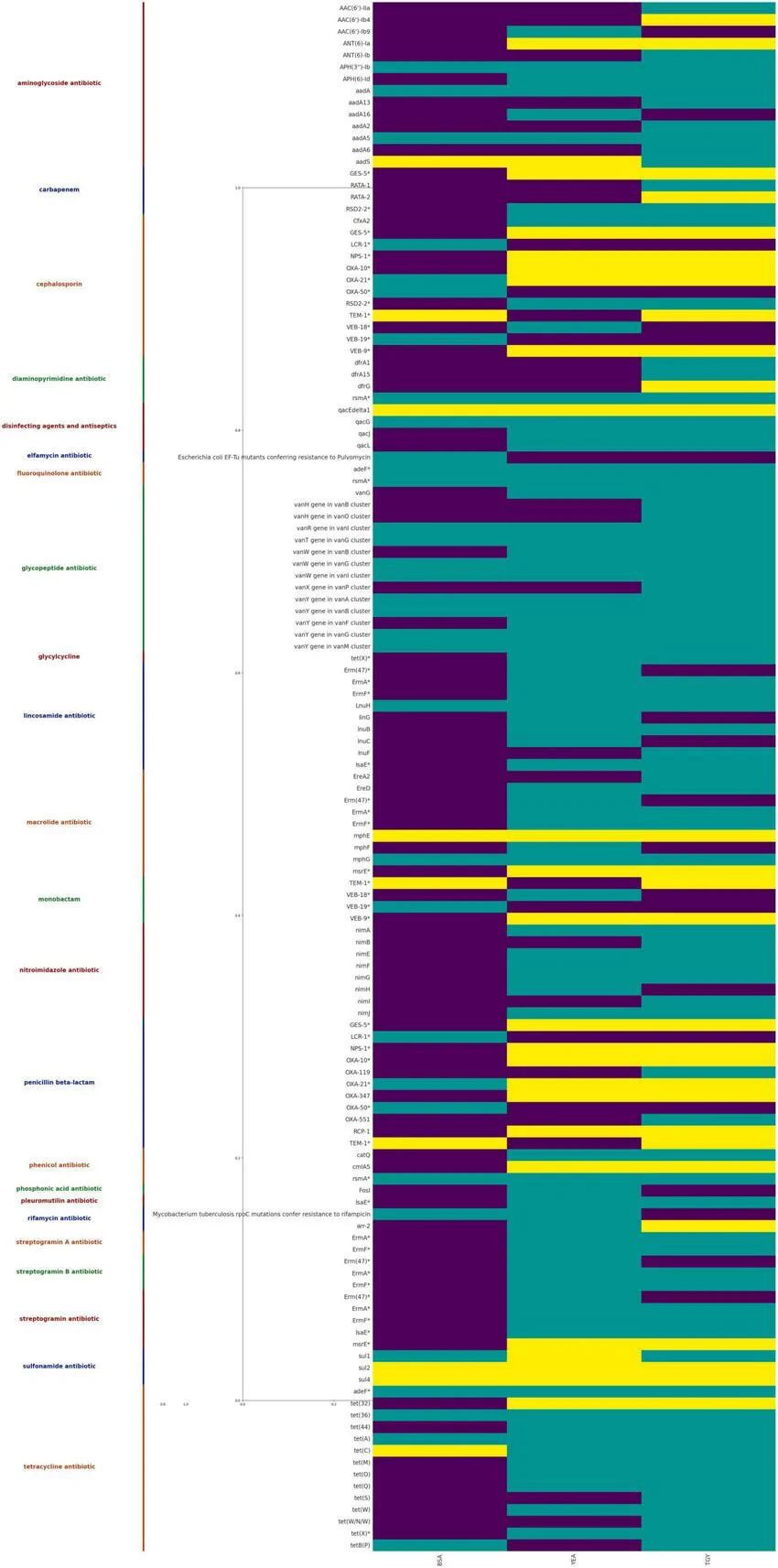
Distribution of antimicrobial resistance (AMR) genes across antibiotic drug classes in Yamuna River sediments. Heatmap showing the presence and confidence of AMR genes detected at the three sampling locations (BSA, TGY, and YEA), classified by major antibiotic classes. Each row represents an individual AMR gene, grouped by drug class (indicated on the left), and each column corresponds to a sampling site. Color coding denotes the confidence of Resistance Gene Identifier (RGI) predictions: yellow indicates a *perfect hit*, teal indicates a *strict hit*, and purple indicates *no hit*. Genes marked with an asterisk (*) appear multiple times due to their association with more than one resistance mechanism category in the antibiotic resistance ontology (ARO).

The distribution of antimicrobial resistance (AMR) genes across the three sampled Yamuna River locations showed apparent differences in gene presence and drug-class representation (Figure 3). AMR genes were detected across a wide range of antibiotic drug classes, with differences in hit confidence levels as indicated by perfect hits (yellow), strict hits (teal), and no hits (purple).

Across all locations, resistance genes associated with β-lactam antibiotics, aminoglycosides, macrolides, tetracyclines, sulfonamides, glycopeptides, fluoroquinolones, disinfecting agents, and antiseptics were prominently represented. Both perfect and strict hits were observed for multiple genes, indicating the presence of high-confidence AMR determinants in the metagenomes. Several genes marked with an asterisk (*) appeared multiple times due to their association with more than one resistance mechanism category in the antibiotic resistance ontology (ARO).

The BSA location exhibited a comparatively lower number of AMR gene hits across many drug classes. Limited strict hits were observed for certain aminoglycoside resistance genes (ad variants) and selected β-lactam resistance genes. Perfect hits were sparse at this site, and several drug classes, such as glycopeptides, macrolides, tetracyclines, and sulfonamides, were represented by few or no high-confidence hits.

The YEA site showed a notable increase in both diversity and confidence of AMR gene detection. Multiple perfect hits were observed for genes conferring resistance to β-lactams, particularly cephalosporins, penicillins, and carbapenems. Aminoglycoside, macrolide, tetracycline, and sulfonamide resistance genes were also frequently detected, with a mixture of perfect and strict hits. Genes associated with resistance to disinfecting agents and antiseptics (e.g., *qac* genes) and to glycopeptides (van gene clusters) were consistently present, indicating broader AMR class representation at this site than in BSA.

TGY exhibited the highest observed representation of AMR drug-class genes based on the detected gene profiles among the three locations. A large number of perfect hits were observed across multiple antibiotic classes, including β-lactams, aminoglycosides, macrolides, tetracyclines, sulfonamides, and phenicols. Tetracycline resistance genes (*tet* variants) were particularly widespread, with both perfect and strict hits dominating the profile. Glycopeptide resistance genes (*van* clusters), rifamycin resistance genes, and resistance determinants linked to disinfecting agents were also consistently detected, reflecting extensive representation of high-confidence AMR genes at this site.

#### 3.4.2 AMR genes based on resistance mechanisms

**FIGURE 4:**
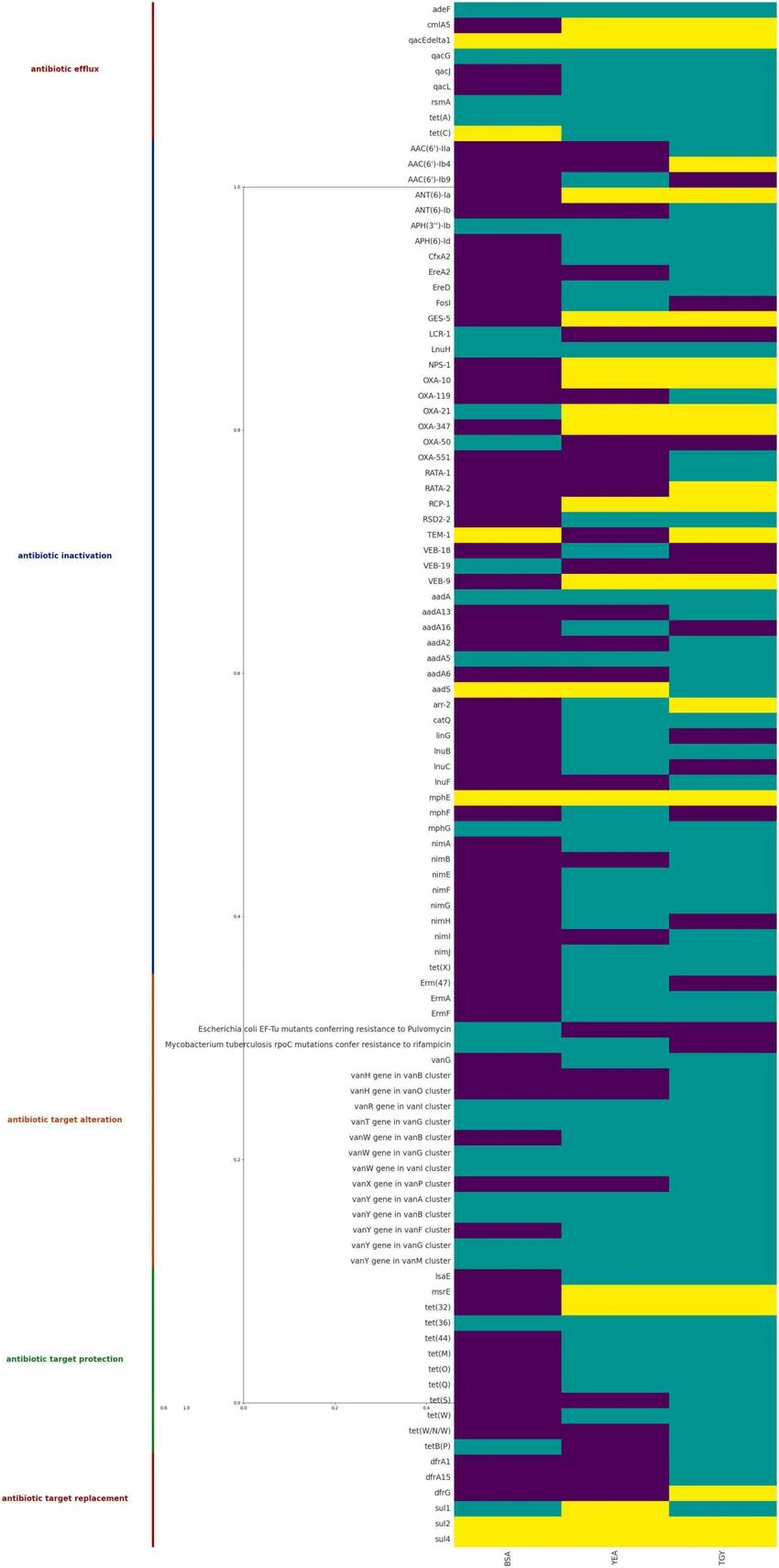
Distribution of antimicrobial resistance (AMR) genes based on resistance mechanisms in Yamuna River sediments. Heatmap illustrating the presence and confidence of AMR genes detected at the three sampling locations (BSA, TGY, and YEA), classified by major resistance mechanisms: antibiotic efflux, antibiotic inactivation, antibiotic target alteration, antibiotic target protection, and antibiotic target replacement. Each row represents an individual resistance gene, and each column corresponds to a sampling site. Color coding indicates the confidence level of Resistance Gene Identifier (RGI) predictions: yellow denotes a *perfect hit*, teal denotes a *strict hit*, and purple denotes *no hit*.

The resistance mechanism–based analysis revealed a diverse repertoire of AMR genes across the three Yamuna River sampling locations encompassing multiple resistance strategies, including antibiotic efflux, antibiotic inactivation, antibiotic target alteration, antibiotic target protection, and antibiotic target replacement (Figure 4). Gene detection varied among sites in both presence and confidence level, represented by perfect hits (yellow), strict hits (teal), and no hits (purple). Several genes marked with an asterisk (*) appeared multiple times because they were associated with more than one resistance mechanism category in the antibiotic resistance ontology (ARO).

Genes associated with antibiotic efflux mechanisms were detected at all three locations, with notable site-wise variation. The YEA and TGY locations showed a higher frequency of perfect and strict hits for efflux-related genes, including multidrug efflux determinants and disinfectant resistance genes (e.g., *qac* family). In contrast, BSA exhibited fewer efflux-related genes, with many showing no-hit patterns and limited strict hits. Overall, efflux mechanisms were more prominently represented in YEA and TGY than in BSA.

The antibiotic inactivation category constituted the largest and most diverse group of resistance mechanism genes across all sites. This category included genes encoding aminoglycoside-modifying enzymes, β-lactamases, chloramphenicol acetyltransferases, and macrolide-modifying enzymes. TGY displayed the highest number of perfect hits, particularly for aminoglycoside-modifying enzymes (*aad*, *aac*, *ant*, *aph* variants) and β-lactamase genes (e.g., *TEM*, *OXA*, *GES* variants). YEA showed a mix of perfect and strict hits across many inactivation genes, whereas BSA showed a predominance of no hits and fewer strict hits, indicating reduced representation of inactivation-based resistance at this site.

Genes involved in antibiotic target alteration, including those associated with glycopeptide resistance (van gene clusters) and ribosomal or enzymatic target modifications, were detected mainly at YEA and TGY. Multiple van cluster genes (vanA, vanB, vanG, vanL, vanM, and vanN associated genes) were consistently observed as strict hits, with occasional perfect hits, particularly at TGY. In contrast, BSA showed limited detection of target-alteration genes, with several van-associated genes showing no-hit patterns.

Resistance genes conferring protection of the antibiotic target, such as ribosomal protection proteins associated with tetracycline resistance (*tet* variants), were predominantly detected at YEA and TGY. TGY exhibited both perfect and strict hits across multiple tet genes, whereas YEA showed mostly strict hits, with occasional perfect hits. BSA demonstrated minimal representation of target protection genes, with most showing no hits.

Genes associated with antibiotic target replacement, including sulfonamide resistance genes (*sul1, sul2, sul4*) and alternative dihydrofolate reductase genes (*dfr* variants), were detected across the sites with marked differences. YEA and TGY showed strong signals, including perfect hits for several *sul* genes and strict hits for *dfr* variants. In contrast, BSA exhibited fewer target replacement genes, with limited strict hits and a higher proportion of no-hit patterns.

#### 3.4.3 VF genes across the three locations

**FIGURE 5:**
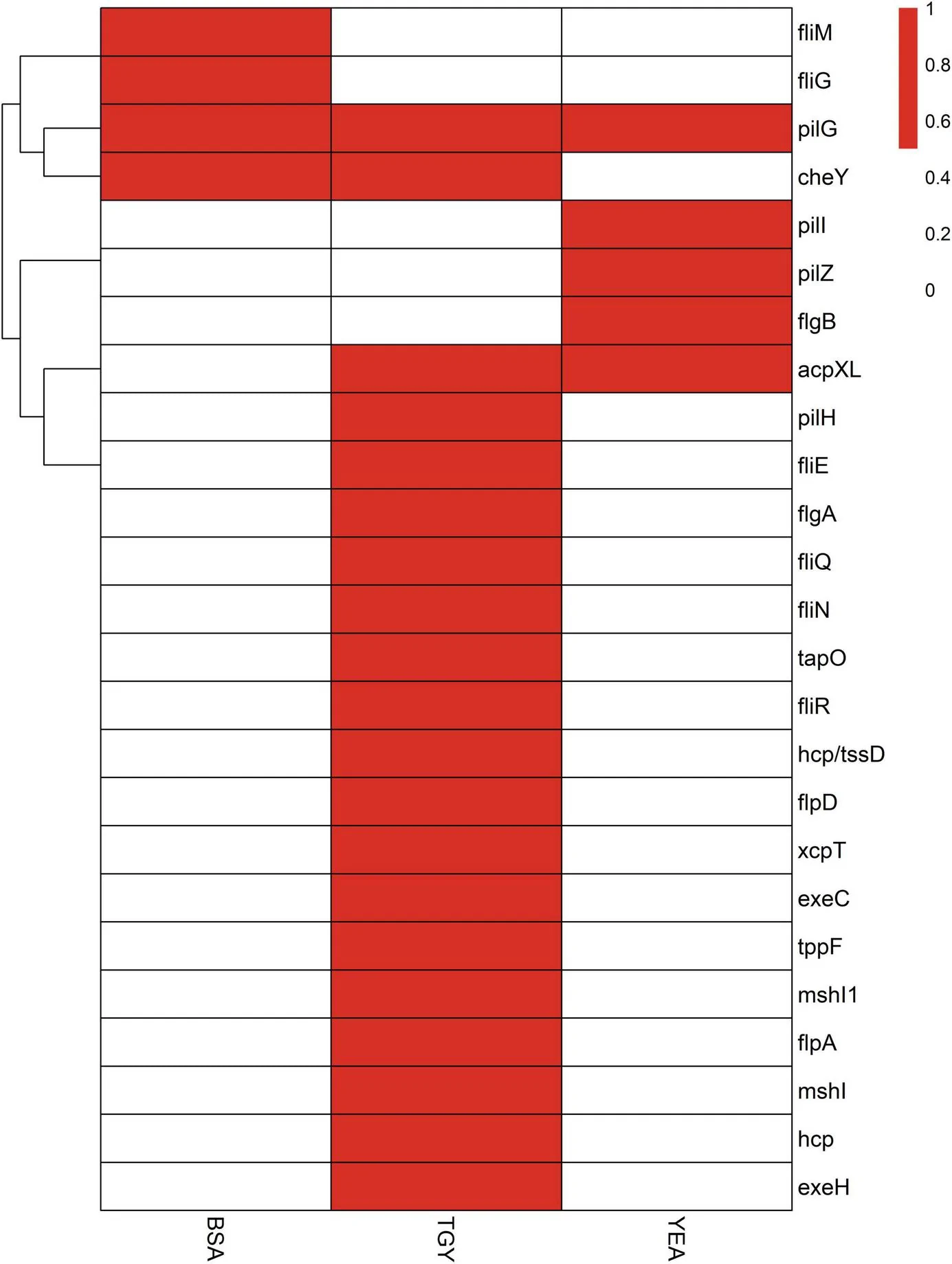
Presence–absence pattern of virulence factor (VF) genes across Yamuna River sediment samples. Heatmap showing the distribution of selected virulence-associated genes detected at the three sampling locations, BSA, TGY, and YEA. Rows represent individual VF genes associated with motility and chemotaxis (e.g., *fli*, *flg*, *che*), pili and fimbriae formation (e.g., *pil*, *flp*, *msh*), and secretion systems including Type II (*xcpT, exeC, exeH*) and Type VI (*hcp/hcp–tssD*) components. Columns correspond to sampling sites. Red cells indicate gene presence, while white cells denote absence.

The presence–absence profiles showed apparent differences in the composition and representation of virulence-associated genes among the three composite sediment samples (Figure 5).

A total set of virulence-associated genes linked to motility, chemotaxis, pili and fimbriae formation, secretion systems, and host interaction was detected across the sites. The distribution was highly uneven, with TGY showing the largest observed VF gene repertoire, followed by YEA, whereas BSA had the smallest observed repertoire. Hierarchical clustering further highlighted distinct VF gene profiles among the three locations.

The BSA site showed limited representation of virulence factor genes, with presence largely restricted to a small subset of motility- and chemotaxis-related genes. Flagellar motor and regulatory genes, such as fliM, fliG, pilG, and cheY, were detected, indicating the presence of basic motility and signal transduction capabilities within the microbial community. However, the majority of genes associated with pili assembly, secretion systems, and virulence-associated transport or effector delivery were absent at this site, reflecting a comparatively low virulence gene repertoire.

TGY exhibited the largest observed repertoire of detected virulence-factor genes, followed by YEA and BSA. A broad suite of genes related to flagellar biosynthesis and function (flgB, fliE, fliN, fliQ, fliR, flgA), type IV pili and fimbrial systems (pilH, flpA, flpD, mshI, mshI1), and chemotaxis and surface attachment was consistently present. Additionally, genes associated with secretion systems, including hcp/hcp–tssD (Type VI secretion system components), xcpT, exeC, and exeH (Type II secretion system components), were uniquely or predominantly detected at this site. This extensive presence suggests a functionally complex microbial assemblage with enhanced motility, colonization, and inter-microbial and host interactions.

The YEA site showed an intermediate virulence gene profile, distinct from both BSA and TGY. Several genes related to pili formation and regulation, such as *pilI* and *pilZ*, were present, along with selected flagellar and secretion-related genes (*flgB*, *acpXL*). However, many VF genes abundantly detected at TGY, particularly those linked to secretion systems and extensive flagellar assembly, were absent in YEA. This indicates moderate virulence potential relative to TGY but greater functional complexity than BSA.

## 4 Discussion

### 4.1 Microbial diversity across the river Yamuna

A comprehensive genus-level taxonomic analysis of microbial communities along the Yamuna River reveals a complex, spatially heterogeneous microbial landscape. Distinct differences in community composition were observed across the three sampling locations: Different from method. A significant proportion of identified taxa were grouped under the “Others” category across all sites. This observation underscores the exceptionally high microbial diversity characteristic of riverine ecosystems, where a substantial fraction of the community comprises low-abundance or phylogenetically unresolved genera (Chen et al., 2020). This pattern is consistent with the recognized phenomenon of a ‘rare biosphere,’ increasingly acknowledged for its crucial contributions to ecosystem resilience and functional redundancy in aquatic environments (Lin Q. et al., 2025; Ye et al., 2023; Zhang et al., 2022). The diversity of rare species has been positively correlated with ecosystem multifunctionality (Zhang et al., 2022), acting as “ecological insurance” to support crucial ecosystem processes (Ye et al., 2023). Furthermore, functional redundancy enhances microbial resilience, particularly in dynamic systems such as streams, by mitigating disturbances (Lin Q. et al., 2025).

The microbial assemblage at the BSA site was characterized by a comparatively higher abundance of *Bacteroidetes* and *Acidobacteria*. These phyla are commonly associated with the degradation of organic matter and adaptation to diverse environmental conditions, including oligotrophic or moderately impacted habitats. *Bacteroidetes* play a key role in the degradation of complex polymers, such as proteins, cellulose, pectin, and chitin, within aquatic environments (Santos-Júnior et al., 2020). While some members of *Bacteroidetes*, like *Arcicella*, are specifically adapted to oligotrophic conditions (Morrison et al., 2017), others demonstrate metabolic versatility by thriving in mesotrophic and eutrophic environments with high nutrient levels (Ma et al., 2016). *Acidobacteria* are recognized as slow-acting decomposers of plant-, fungal-, and insect-derived organic polymers (Sikorski et al., 2022) and are typically considered oligotrophic, often being more abundant in environments with low organic carbon availability (Cuevas et al., 2024; Kalam et al., 2020). The prevalence of unclassified representatives within these phyla suggests the presence of locally adapted or underexplored lineages, potentially indicative of baseline riverine conditions or limited anthropogenic influence. Furthermore, the marginal representation of well-characterized genera such as *Flavobacterium* and *Clostridium* at BSA supports the notion of a less specialized, functionally conservative community structure at this site.

In contrast, the TGY site exhibited a discernible taxonomic shift, marked by an increased representation of *Firmicutes* and *Lentisphaerae*-associated taxa, concomitant with a relative reduction in *Acidobacteria*. Members of *Firmicutes* are frequently associated with enhanced metabolic versatility, stress tolerance, and the ability to persist under fluctuating environmental conditions (Filippidou et al., 2016; Schreckinger et al., 2021; Shahraki et al., 2021; White et al., 2018). Many *Firmicutes* genera are endospore-forming, which provides improved persistence against stressors such as drying, desiccation, and osmotic stress, due to their gram-positive cell wall type and spore-forming capabilities (Filippidou et al., 2016; Sabater et al., 2016; Schreckinger et al., 2021). Their diverse metabolic capabilities and resistance to oxygen limitation further contribute to their adaptability (Shahraki et al., 2021; White et al., 2018). Consequently, the observed taxonomic profile at TGY suggests a microbial community adapted to more dynamic or nutrient-enriched environments. The persistent, albeit low-level, presence of *Paludibacter*, *Flavobacterium*, and *Clostridium* across sites indicates the conservation of core functional groups despite shifts in their relative abundances.

The microbial community at YEA showed broad similarities to that of TGY but also exhibited distinct compositional nuances. Notably, an increased contribution of Betaproteobacteria was observed, alongside Bacteroidetes, Chloroflexi, and Deltaproteobacteria. Betaproteobacteria are frequently implicated in critical nutrient cycling processes and exhibit rapid responses to environmental gradients and nutrient additions (Zadjelovic et al., 2023). Their presence in freshwater bodies is well-documented, and they play key roles in carbon and nitrogen cycling (Zadjelovic et al., 2023). Their rapid response to nutrient fluctuations indicates an adaptation to dynamic aquatic conditions (Balmonte et al., 2016; Ma et al., 2016). This suggests that YEA may represent a transitional ecological state influenced by both upstream baseline river conditions and localized inputs. The consistency of low-abundance genera between YEA and TGY suggests shared environmental drivers, while subtle variations in relative abundance likely reflect site-specific selective pressures.

Collectively, these taxonomic patterns indicate that while the Yamuna River harbors a broadly conserved microbial framework, the observed site-level profiles are consistent with a potential influence of localized environmental conditions on the relative dominance of specific microbial groups. Riverine microbial communities are known to be diverse, dynamic, and spatially heterogeneous, with spatial variables often being dominant factors driving community diversity (Gweon et al., 2021; Liu et al., 2022). The observed shift from relatively greater representation of Acidobacteria at BSA toward increased representation of Firmicutes, Lentisphaerae, and Betaproteobacteria at TGY and YEA is consistent with spatial heterogeneity in microbial community composition; however, this pattern should be considered exploratory because within-site replication was not available (Liu et al., 2022; Xue et al., 2025). These findings emphasize the critical importance of fine-scale taxonomic analyses for a comprehensive understanding of microbial ecological dynamics in riverine systems, as class-level patterns may fail to capture the nuances of individual members (Ruiz-González et al., 2015). Such detailed analyses provide a foundational basis for linking community composition to functional potential and environmental stressors along the Yamuna River.

A limitation of the present study is the absence of independent biological replicates within each sampling location. Although five sediment subsamples were collected approximately 250 m apart at each site, these subsamples were pooled to generate a single composite sample, resulting in one independent metagenomic sample per location. Consequently, within-site variability could not be quantified, and inferential statistical comparisons among locations, including estimation of variance or formal testing of side effects, were not possible. The observed differences in microbial diversity, community composition, AMR genes, and virulence factors should therefore be interpreted as exploratory site-level patterns rather than population-wide spatial effects. Future studies incorporating multiple independent biological replicates per site, preferably across seasons and environmental gradients, will be necessary to quantify within-site variability and statistically validate the spatial patterns observed here.

### 4.2 Anthropogenic gradients drive multiclass AMR enrichment in the Yamuna river

A comprehensive drug classification-based analysis revealed significant spatial heterogeneity in the distribution and prevalence of antimicrobial resistance genes along the Yamuna River. A clear gradient was observed in all three locations. This observed pattern underscores the critical influence of localized environmental conditions and anthropogenic pressures in shaping the riverine resistome (Keely et al., 2022; Serrana et al., 2025; Wang J. et al., 2022).

An important consideration is that not all metagenomic sequences could be resolved to the species level. This limitation does not preclude the identification of ARGs because RGI identifies resistance determinants from predicted protein sequences through comparison with curated CARD resistance models. However, the absence of species-level resolution limits direct attribution of individual ARGs to specific bacterial hosts. Accordingly, the present analysis supports the occurrence and functional classification of resistance determinants within the metagenomic community but does not establish which particular bacterial species carry each ARG. Linking ARGs to their microbial hosts would require higher-resolution approaches, such as contig-level taxonomic binning, metagenome-assembled genomes, or long-read sequencing, ideally complemented by plasmid/mobile-element analysis.

The widespread detection of AMR genes associated with β-lactams, aminoglycosides, macrolides, tetracyclines, sulfonamides, fluoroquinolones, glycopeptides, and disinfectants across all sampling sites indicates that the Yamuna River harbors a diverse, multi-class resistome (Amarasiri et al., 2022; Marti et al., 2013; Xiong et al., 2014). The presence of both perfect and strict hits for several resistance genes suggests that a substantial fraction of these determinants closely aligns with curated resistance models, thereby reflecting well-established and potentially functional AMR genes rather than distant homologs or spurious matches. This indicates the integrity and functional relevance of the identified resistance elements within the aquatic environment. The recurrence of certain genes across multiple resistance mechanism categories further emphasizes the functional versatility of key resistance determinants within the overall antimicrobial resistome.

The lower observed normalized representation of AMR determinants at BSA is consistent with a lower apparent resistome burden in the sampled composite sediment; however, the absence of biological replication prevents inference about site-wide AMR abundance. The greater normalized representation of selected resistance determinants at TGY and YEA is consistent with stronger apparent resistome enrichment in these samples, although environmental exposure cannot be established as a causal factor from the present design (Chopyk et al., 2020; Kraemer et al., 2022). The predominance of no-hit patterns and the scarcity of perfect hits across most drug classes at BSA imply that resistance genes at this site may be present at lower abundance, potentially fragmented, or less evolutionarily optimized for resistance. Such profiles are characteristic of background or baseline resistomes, commonly found in less-impacted freshwater environments (Kraemer et al., 2022).

In contrast, the YEA site exhibited a marked expansion in both the diversity and confidence of AMR gene detection, particularly for clinically important drug classes such as β-lactams, aminoglycosides, macrolides, tetracyclines, and sulfonamides. The identification of multiple perfect hits for β-lactam resistance genes, including those associated with cephalosporins, penicillins, and carbapenems, is particularly concerning given their clinical relevance (Ott et al., 2021). The consistent detection of *qac* genes (often associated with disinfectants and biocides) and *van* gene clusters (conferring glycopeptide resistance) further suggests co-selection pressures driven by the widespread use of disinfectants and glycopeptides. These agents are known to promote the persistence and enrichment of multidrug resistance in aquatic systems (Amarasiri et al., 2022).

The TGY location emerged as a hotspot of AMR gene diversity and confidence, exhibiting the highest abundance of perfect hits across nearly all major antibiotic classes. The extensive representation of tetracycline resistance genes (tet variants), alongside robust signals for β-lactams, aminoglycosides, macrolides, sulfonamides, and phenicols, points to strong and sustained selective pressures at this site (Abdelall et al., 2020; Xiong et al., 2014). The consistent detection of glycopeptide (van cluster) and rifamycin resistance genes further indicates the accumulation of resistance determinants typically associated with high antibiotic exposure environments. Such profiles are characteristic of heavily impacted river stretches receiving complex mixtures of municipal, hospital, agricultural, and industrial discharges (Lee et al., 2023; Marti et al., 2013; Wang Q. et al., 2022; Xiong et al., 2014; Yang et al., 2025).

The observed progressive enrichment of perfect hits from BSA to YEA and TGY suggests not only an increase in AMR gene abundance but also a shift toward resistance genes with higher sequence identity to known functional resistance determinants (Marti et al., 2013; Ott et al., 2021). This pattern implies active selection and stabilization of AMR genes at downstream or more affected sites, thereby enhancing their likelihood of expression, persistence, and potential for horizontal gene transfer within microbial communities (de Oliveira et al., 2024; Yang et al., 2025). Drug classes such as β-lactams, tetracyclines, macrolides, and sulfonamides, which showed the strongest contrasts among sites, are widely used in human medicine, veterinary practice, and agriculture. This reinforces the direct link between widespread antibiotic usage patterns and the structuring of environmental resistomes (Jiang et al., 2022; Keely et al., 2022; Wang J. et al., 2022).

An important avenue for future interpretation of riverine resistome patterns is the integration of molecular measurements with spatially explicit environmental observations. Multi-temporal remote-sensing approaches can provide information on changes in ecological condition, land degradation, vegetation, surface characteristics, and catchment stability over spatial and temporal scales that cannot be captured by individual field sampling campaigns. In the Ganga basin, multi-temporal LANDSAT analysis has demonstrated substantial spatio-temporal variability in ecological stability (Bhattacharjee et al., 2024). Integrating such remotely sensed environmental indicators with metagenomic measurements of microbial communities, ARGs, and virulence-associated genes could help distinguish persistent environmental patterns from short-term or site-specific microbial responses.

### 4.3 Mechanism-specific structuring of the Yamuna river resistome

A detailed resistance mechanism-based profiling of Yamuna River metagenomes reveals a complex and spatially structured AMR landscape, where both the diversity of resistance strategies and the confidence of gene detection increase progressively from the BSA to the YEA and TGY. This pattern highlights the multifaceted nature of environmental resistomes and their capacity to counteract antimicrobial pressure through complementary strategies (Bobate et al., 2023; Danner et al., 2019; Peterson and Kaur, 2018).

Among the detected mechanisms, antibiotic efflux was most prominently observed in YEA and TGY, particularly through multidrug efflux systems and disinfectant resistance genes, such as those of the *qac* family. Efflux systems confer broad-spectrum resistance by actively expelling antimicrobials from the bacterial cell, thereby preventing them from reaching intracellular targets (Bobate et al., 2023; Bogri, 2024; Zhao et al., 2023). These mechanisms are frequently selected under chronic exposure to antimicrobials and biocides, including metals, which can co-select for and maintain resistance genes (Nguyen et al., 2023; Sidibe et al., 2023). Their enrichment at YEA and TGY suggests sustained exposure to mixed chemical stressors, including residual antibiotics and heavy metals, which can exert selective pressure even at sub-inhibitory concentrations (Flores-Vargas et al., 2024; Wen et al., 2016). The comparatively weak representation of efflux genes at BSA is consistent with lower selective pressure at this site, potentially reflecting lower exposure to such stressors.

Antibiotic inactivation emerged as a dominant resistance strategy, particularly at TGY, where a high number of perfect hits were detected for aminoglycoside-modifying enzymes and β-lactamases. Enzymatic inactivation provides highly effective and often drug-class-specific resistance by chemically modifying the antibiotic molecule, thereby neutralizing its antimicrobial activity before it can reach its targets (Markley and Wencewicz, 2018; Zhao et al., 2023). The dominance of inactivation at TGY indicates strong and persistent selection for such effective mechanisms (Lin J. et al., 2025; Zhu et al., 2025). The enrichment of β-lactamase families, including *TEM* and *OXA*, is notable given their widespread association with clinically important resistance in both human and veterinary medicine (Piotrowska et al., 2019). The global distribution of β-lactamases across diverse environments and bacterial phyla further emphasizes their widespread presence (Gholipour et al., 2024). The increasing spread of extended-spectrum β-lactamase-producing Gram-negative bacteria is considered a significant public health threat (Moreira Soares de Souza et al., 2021). This suggests a substantial overlap between environmental and clinically relevant resistomes at impacted river sites, particularly those affected by wastewater treatment plants, which are known anthropogenic hotspots for the enrichment and transfer of such genes (Kim and Cha, 2021; Wang J. et al., 2022). The lower prevalence of inactivation genes at BSA supports the interpretation that it is a site less influenced by intensive antimicrobial inputs.

Genes associated with antibiotic target alteration, particularly those belonging to glycopeptide resistance *van* clusters, were largely restricted to YEA and TGY. Target alteration mechanisms involve modifications to the antibiotic’s binding site through point mutations or enzymatic modifications, such as ribosomal methylation or alterations in peptidoglycan precursors (Guillén-Chable et al., 2022; Jahn, 2019; Peterson and Kaur, 2018). The detection of multiple *van* gene variants implies the presence of intact resistance operons or partial clusters capable of modifying antibiotic targets. These mechanisms are often associated with high-level resistance and are typically maintained under strong selective pressure (Guillén-Chable et al., 2022).

Similarly, antibiotic target protection mechanisms, dominated by tetracycline ribosomal protection proteins (tet variants), were substantially enriched at YEA and TGY. Target protection prevents the antibiotic from binding to its target without chemically altering or inactivating the drug itself (Bogri, 2024; Jahn, 2019). The co-occurrence of perfect and strict hits for multiple tet genes at TGY suggests both diversity and evolutionary optimization of target protection strategies, complementing other resistance pathways and enhancing overall resistance resilience (Guillén-Chable et al., 2022).

Antibiotic target replacement mechanisms, including *sul* and *dfr* genes, were also strongly represented at YEA and TGY. These genes encode alternative metabolic enzymes that bypass antibiotic-sensitive targets, allowing continued cellular function in the presence of antimicrobial compounds (Jahn, 2019). The detection of perfect hits for multiple *sul* genes indicates well-established resistance determinants that are frequently associated with mobile genetic elements, raising concerns about their potential for horizontal transfer within aquatic microbial communities (Peterson and Kaur, 2018).

Collectively, the co-dominance of efflux and inactivation mechanisms, coupled with the substantial presence of target-altering, protective, and replacement strategies at YEA and TGY, reflects a highly adapted resistome shaped by sustained and heterogeneous selection pressures. In contrast, the limited representation of all resistance mechanisms at BSA suggests a background resistome with lower functional complexity and reduced selective reinforcement. The observed site-level gradient in resistance-mechanism diversity and prediction confidence is consistent with potential differences in localized environmental conditions among the sampled locations (Jiang et al., 2022; Kubera, 2021). However, because only one composite sample was analyzed per location, these observations should be regarded as exploratory and cannot establish statistically supported spatial gradients or causal relationships with environmental pressures. Heavily impacted river stretches, therefore, serve as critical reservoirs for diverse and potentially transferable resistance mechanisms (Jiang et al., 2022; Kim and Cha, 2021; Wang J. et al., 2022).

### 4.4 Emergence of virulence hotspots in Yamuna river stretches

A spatially resolved analysis of virulence factor genes along the Yamuna River reveals pronounced site-specific structuring of virulence potential, with a clear gradient from the BSA, which exhibits the lowest, through the YEA, which shows intermediate, to the TGY, which demonstrates the highest virulence potential. This pattern mirrors the functional complexity of microbial communities at each location and underscores the strong influence of local environmental conditions and anthropogenic impacts on the maintenance and distribution of virulence-associated traits in riverine ecosystems (Al Haj Ishak Al Ali et al., 2023; Cardenas-Alegria et al., 2024).

The limited VF gene repertoire at BSA, largely restricted to core motility and chemotaxis genes such as *fliM, fliG, pilG*, and *cheY*, suggests the dominance of microorganisms primarily equipped with basic locomotion and environmental sensing capabilities, rather than specialized host interaction or competitive colonization mechanisms. Motility and chemotaxis are crucial for bacterial growth and survival in aquatic environments, enabling movement toward nutrients and away from toxins (Fouts et al., 2016; Matanza and Osorio, 2018; Romero et al., 2021). These genes are widely conserved across aquatic bacteria and primarily facilitate survival, dispersal, and nutrient foraging (Murphy et al., 2021; Romero et al., 2021; Talagrand-Reboul et al., 2017). The absence of genes linked to pili assembly, secretion systems, and effector delivery indicates a comparatively low virulence potential, suggesting that BSA may represent a background or baseline microbial community with reduced selective pressure for virulence-associated functions (Adade et al., 2022).

In contrast, TGY exhibited a highly enriched and diverse virulence gene profile, encompassing multiple systems involved in motility, adhesion, and secretion. The extensive presence of flagellar biosynthesis genes (*flg* and fli families) indicates enhanced motility and surface colonization capacity, which are vital for adapting to environmental changes (De Maayer et al., 2020a, b). Flagella also play crucial roles in bacterial adhesion and biofilm formation (Jian et al., 2016; Khan et al., 2020; Wang et al., 2015). The detection of type IV pili and fimbrial genes (*pil*, *flp*, *msh* families) points to strong attachment capabilities, biofilm formation, and surface-associated lifestyles (Lin et al., 2022; Shagieva et al., 2020; Wang Q. et al., 2022). Notably, the consistent detection of Type VI (hcp/*hcp*–*tssD*) and Type II (*xcpT*, *exeC*, *exeH*) secretion system components highlights advanced mechanisms for interbacterial competition, environmental persistence, and potential host interaction (Barger et al., 2020; Nikolić et al., 2023; Yang et al., 2021; Yin et al., 2024). These secretion systems enable bacteria to deliver toxins into target cells, influencing competitive advantages and pathogenicity (Alcoforado Diniz et al., 2015). Together, these features reflect a functionally complex microbial assemblage with elevated ecological competitiveness and virulence-associated capabilities, characteristics often associated with highly impacted or nutrient-rich aquatic environments (Cardenas-Alegria et al., 2024).

The intermediate VF gene profile observed at YEA suggests a transitional microbial community. It retains some capacity for adhesion and surface-associated growth via pili-related genes (pilI, pilZ) and selected flagellar genes. Flagellar genes contribute to motility, which can facilitate colonization and interaction capabilities (De Maayer et al., 2020a; Yang et al., 2023), though YEA lacks the full complement of secretion and extensive motility systems observed at TGY. This intermediate profile positions YEA between the baseline state observed at BSA and the highly complex virulence landscape of TGY.

Collectively, the observed gradient in VF gene diversity and composition highlights how riverine microbial communities can shift from primarily survival-oriented strategies to more complex interaction- and competition-oriented lifestyles along spatial or environmental gradients (Petit et al., 2017). The enrichment of genes related to adhesion, motility, and secretion at TGY suggests an increased capacity for biofilm formation, inter-microbial antagonism, and potential host association (Zadjelovic et al., 2023), raising important considerations for microbial ecology and public health in impacted river stretches (Lepuschitz et al., 2019; Nogueira et al., 2021). These findings emphasize the value of virulence gene surveillance in aquatic environments as an indicator of ecosystem disturbance and microbial functional shifts, informing on ecological site quality and potential public health risks (Abioye et al., 2023; Deng et al., 2023; Mavhungu et al., 2023).

## 5 Conclusion

This study provides an exploratory metagenomic assessment of microbial diversity, antimicrobial resistance (AMR), and virulence factors in sediments collected from three locations of the Yamuna River near Agra, India, offering critical insights into the ecological and public health dimensions of riverine pollution. By integrating high-throughput shotgun metagenomics with robust taxonomic, functional, and diversity analyses, reveal apparent differences in microbial community structure and functional potential among the three sampled locations, providing a basis for further investigation of spatial patterns in the Yamuna River. The progressive increase in microbial diversity, AMR gene abundance, resistance mechanism complexity, and virulence-associated traits from BSA to TGY and YEA underscores the strong influence of localized anthropogenic pressures on sediment microbiomes. The enrichment of clinically relevant AMR determinants, particularly β-lactamases, tetracycline resistance genes, and multidrug efflux systems, along with diverse virulence-related genes at impacted sites, highlights the role of river sediments as reservoirs and amplification hubs for environmentally persistent resistance and pathogenic traits. The co-occurrence of AMR genes and virulence factors further raises concerns regarding their potential mobilization and transfer within microbial communities, with implications for ecosystem health and human exposure. Overall, this study establishes a critical baseline for environmental resistome and virulome surveillance in the Yamuna River and underscores the need to integrate microbial genomic monitoring into river management and pollution mitigation strategies. Such evidence-based approaches are essential for safeguarding the integrity of aquatic ecosystems and minimizing emerging public health risks in contaminated riverine environments.

## Data Availability

The metagenomics data, obtained from our investigation, have been submitted to the NCBI SRA and can be accessed using the accession numbers SRR16085952 (BSA), SRR16085951 (TGY), and SRR16085950 (YEA).
