# Supplementary material for: Metagenomic insights into antibiotic resistance genes and virulence factors in sediments of river Yamuna
Source: Front Microbiol. 2026 Aug 28;17:1938385. doi: 10.3389/fmicb.2026.1938385 (PMC13561774; doi:10.3389/fmicb.2026.1938385)
Supplement: Supplementary file 1 [file Table_1.DOCX]

**Table S1.** Physicochemical parameters of water and sediment at different sampling sites

|  | **Parameters** | **BSA** | **TGY** | **YEA** |
| --- | --- | --- | --- | --- |
| **Water** | DO (ppm) | 4.8 | 4.6 | 2.8 |
|  | pH | 7.96 | 7.1 | 7.4 |
|  | TDS (ppm) | 1606 | 782 | 754 |
|  | ORP (mV) | 122.6 | 238.4 | 29.9 |
|  | Specific conductivity (µS/cm) | 1050 | 950 | 859 |
|  | Salinity (psu) | 0.84 | 0.96 | 0.86 |
|  | BOD (ppm) | 23.9 | 6.7 | 4.2 |
|  | COD (ppm) | 13.2 | 32.9 | 18.9 |
| **Sediment** | pH | 8.1 | 7.3 | 7.56 |
|  | Specific conductivity (µS/cm) | 282 | 363 | 383 |
|  | Total nitrogen (%) | 0.05 | 0.05 | 0.06 |
|  | Available phosphate (mg/100 g) | 0.38 | 1.75 | 0.89 |
|  | Organic carbon (%) | 0.35 | 0.12 | 0.48 |
